# Study of the Microstructure Characterization and In Situ Observation of Crack Propagation in TC4/Al_3_Ti Metal–Intermetallic Laminated Composites

**DOI:** 10.3390/ma19061052

**Published:** 2026-03-10

**Authors:** Yuzhong Miao, Yan Shi, Wenbo Wang, Xuefeng Ding, Shoubin Zhang

**Affiliations:** 1 Ningbo Institute of Technology (NIT), Beihang University, Ningbo 315000, China; yzmiao@buaa.edu.cn (Y.M.);; 2 School of Material Science and Engineering, Beihang University, Beijing 100191, China

**Keywords:** MIL composites, KAM map, EBSD, in situ tensile test, toughening mechanism

## Abstract

TC4/Al_3_Ti metal–intermetallic laminated (MIL) composites were fabricated by the vacuum hot-pressing process at 650 °C. The microstructure characteristics, i.e., grain boundary distribution, crystallographic orientation and Kernel Average Misorientation (KAM) map, were analyzed using EBSD. Meanwhile, the distribution of local strain and the fracture behavior of TC4/Al_3_Ti MIL composites during tensile process were determined by Digital Image Correlation (DIC) and in situ tensile experiments, respectively. Results show that the TC4/Al_3_Ti interfaces are well bonded and exhibit a distinct wavy morphology. The obvious Kirkendall pores and centerline are observed within the central region of the Al_3_Ti layer. The texture components of (10-10) <0001> and (11-20) <10-10> are predominant in the TC4 layers; (100) <001> and (110) <001> are observed in the Al_3_Ti layer. Additionally, the average geometrically necessary dislocation (GNDs) density is 2.53 × 10^14^ m^−2^ in the TC4 layer, whereas it is 1.74 × 10^14^ m^−2^ in the Al_3_Ti layer. In the tensile test, the fracture resistance of TC4/Al_3_Ti MIL composites is significantly improved by the plastic deformation of the TC4 layers and the suppression of crack-tip instability. It is found that the extrinsic toughening mechanisms contain crack deflection, crack blunting, crack bridging, multiple cracking modes, and the plastic deformation of ductile TC4 layers in TC4/Al_3_Ti MIL composites. The real-time observation technique may provide more complete insights into the relationship between fracture behavior and enhanced toughness.

## 1. Introduction

Metal–intermetallic laminate (MIL) composites are regarded as a potential replacement for monolithic materials due to their ability to stack and overcome the strength–ductility trade-off dilemma [1,2]. TC4/Al_3_Ti MIL composites in particular, with low density, high strength-to-weight ratio, and high melting point, possess satisfactory structural efficiency for aerospace and defense applications, used as TC4/Al_3_Ti laminated armors in armored vehicles to achieve lightweight anti-penetration performance [3]. However, the occurrence of premature crack initiation and delamination is detrimental to mechanical performance, arising from stress concentration effects induced by defects (e.g., Kirkendall voids and centerline [4]), microstructure heterogeneity [5] and tensile stress sensitivity during the tensile process. In general, most research on fracture behavior is benefited by the observation of fracture morphology to investigate fracture characteristics and mechanisms. It is a bit limited in exploring the evolution of crack propagation and external toughening mechanisms during plastic deformation. In fact, in situ experimental technology is found to effectively reveal crack evolution and other fracture behaviors during tensile processing [6].

MIL composites were previously fabricated by the explosive welding method, and crack initiation and growth were observed during an in situ tensile test [7,8]. Additionally, five-ply ASS/Al/Mg/Al/ASS laminated composites were prepared by hot-rolling, and the complete fracture processes were characterized by in situ investigation [9]. Nevertheless, the reported in situ tensile studies mainly focus on fracture behavior and mechanical properties at present, ignoring the influence of original microstructure characteristics on fracture behavior. It is well established that crack propagation is affected by the driving force at the crack tip (*J*_tip_) and the resistance inherent to the microstructure (*R*), and the latter is mainly determined by the original microstructure characteristics [10,11]. Moreover, the primary fracture location of TC4/Al_3_Ti MIL composites mainly originates from the Al_3_Ti layers, whereas the distribution and evolution of crack propagation near the TC4/Al_3_Ti interfaces have not been meticulously studied in the tensile test. In addition, enhanced toughness is achieved through secondary cracks and crack-tip convolution in the fracture behavior [12], which is essentially regulated by the deformation incompatibility between the Al_3_Ti and TC4 layers. Thus, crack propagation is not only related to the fracture resistance of the Al_3_Ti layer but also to the deformation of the adjacent ductile layer, which is regulated by microstructure heterogeneity, and the latter is effective.

In addition, the presence and types of interfaces significantly affect the mechanical behavior of TC4/Al_3_Ti MIL composites. The role of the TC4/Al_3_Ti interfaces is found to be associated with stress regulation, strengthening, and toughening mechanisms in MIL composites. For example, hetero-deformation-induced (HDI) hardening is attributed to the distribution and evolution of strain gradients in the interface-affected zone (IAZ) [13]. Additionally, the fracture behavior varies from interfacial bonding strength [14]. It has been reported that delamination occurs prior to interface cracks in weak interfaces, whereas delamination occurs after crack propagation in well-bonded interfaces [15]. Thus, local failure of TC4/Al_3_Ti interfaces may lead to premature fracture of TC4/Al_3_Ti MIL composites during tensile testing. However, the fracture behavior of MIL composites near the TC4/Al_3_Ti interfaces has not been emphasized in some in situ tensile test research. For example, delamination at the TC4/Al_3_Ti interface, a local failure mode, is one of the tensile failure features in the microscopic fracture morphology. It is unclear whether delamination leads to final tensile failure or occurs concurrently with it during tensile fracture. It is hoped to determine whether local delamination leads to final tensile failure via fracture in the in situ tensile test in this study. Therefore, it is necessary and novel to explore the crack-propagation and toughening mechanisms at TC4/Al_3_Ti interfaces in TC4/Al_3_Ti MIL composites, accounting for microstructure heterogeneity.

Meanwhile, the microstructure and defect characteristics are related to the diffusion kinetics and reaction process in the Ti-Al system, which is accompanied by the migration and aggregation of metallic atom vacancies. At temperatures below the melting point of Al, the diffusion coefficient of Al atoms exceeds that of Ti atoms, indicating that Al diffusion dominates the Ti-Al reaction. As reported in the literature [16], the Al_3_Ti phase primarily nucleates and grows on the Al side, leading to migration of the reaction front and accumulation of oxides. As diffusion proceeds, numerous vacancies form behind the reaction front and become increasingly difficult to fill, leading to the formation of Kirkendall voids. The study by Fukutomi et al. [17] also confirmed that the lack of atomic supply is the main cause of Kirkendall void formation in the Al_3_Ti phase. Consequently, the Al_3_Ti layer occupies the original position of the Al foil after hot-pressing, resulting in Kirkendall voids in the central region of the Al_3_Ti layer.

Based on the above scientific issues regarding microstructure heterogeneity and crack propagation in TC4/Al_3_Ti MIL composites, this paper established quantitative statistics of local microstructure characteristics and an in situ tensile test study combined with fracture mechanics theory. In detail, TC4/Al_3_Ti metal–intermetallic laminated (MIL) composites were fabricated via vacuum hot-pressing. On the aspects of microstructure characteristics, the grain boundary distribution, crystallographic orientation and Kernel Average Misorientation (KAM) map were analyzed using EBSD. In the investigation of mechanical behavior, in situ characterization of crack propagation in TC4/Al_3_Ti MIL composites is conducted under tensile loading, including the evolution of cracks in Al_3_Ti layers and near TC4/Al_3_Ti interfaces. Meanwhile, the deformation behavior of ductile TC4 layers is investigated after local failure of the Al_3_Ti layer, and the distribution and evolution of local strain during plastic deformation are assessed via Digital Image Correlation (DIC).

## 2. Experimental Procedure

### 2.1. Material Fabrication

Commercial TC4 foils(Henglongtai, Baoji, China)and Al1060 foils (Pangji, Shanghai, China) were chosen as raw materials, and the chemical compositions of them are given in Table 1. It is noted that such compositional data is typically provided by raw material suppliers as a general, standardized reference and does not represent a unique or proprietary dataset. To ensure the material’s performance, maximum limits have been set for each impurity. Firstly, they were immersed in 5 vol % HF and 5 vol % NaOH for 3 min to remove oxides and impurities, respectively. Then they were cleaned in an ultrasonic cleaner for 3 min. The TC4/Al_3_Ti MIL composites, prepared by stacking “TC4-Al-TC4-Al-TC4” layers for in situ tensile testing, had 35 layers in total, including 18 pieces of TC4 foils and 17 pieces of Al foils, with the top and bottom layers stacked with TC4 foils. Finally, they were sintered in a ZT-40-21Y vacuum hot-pressing sintering furnace (Chenhua Technology Co., Ltd., Shanghai, China).

**Figure 1:**
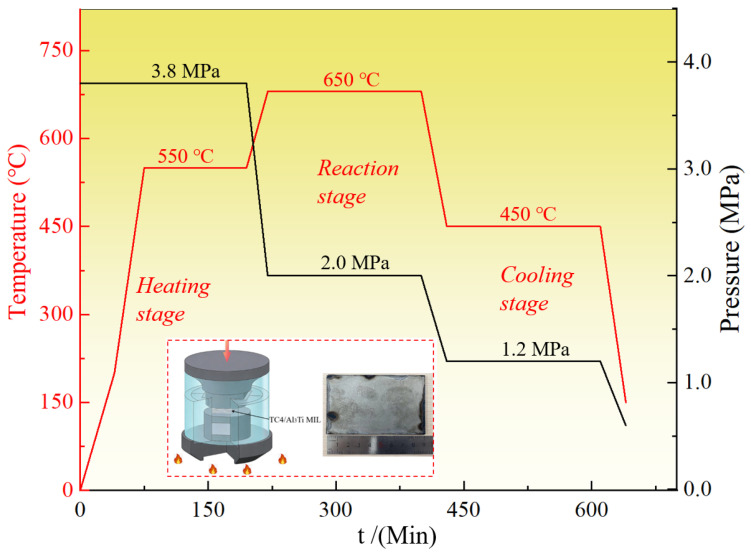
Schematic diagram of vacuum hot-pressing processes and parameters for TC4/Al_3_Ti MIL composites.

As shown in Figure 1, the heating, reaction, and cooling stages are included in the typical vacuum hot-pressing process. Firstly, the furnace temperature was raised to 550 °C at a heating rate of 5–10 °C/min and maintained for 120 min under 3.8 MPa pressure to conduct a partial reaction. The next stage was to elevate furnace temperature to 650 °C for 240 min under 2.0 MPa pressure to allow sufficient reaction and well-debonding. Eventually, the annealing temperature was adjusted to 450 °C for 180 min to reduce the internal residual stress, and the sample was cooled to ambient temperature.

### 2.2. Characterization Methods

**Figure 2:**
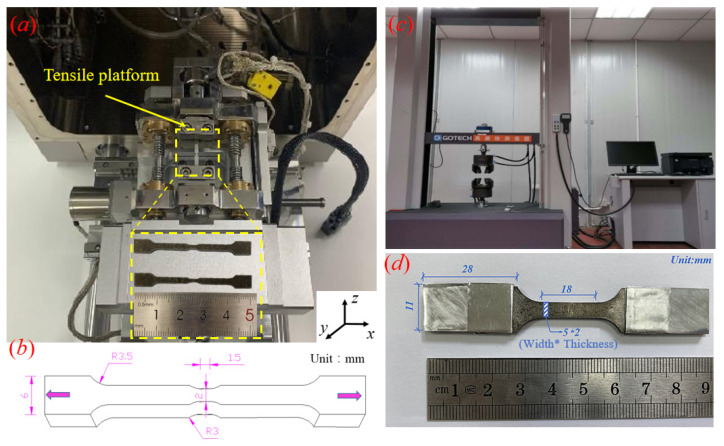
The illustration of (**a**) in situ tensile platform and stretching specimens. (**b**) Schematic of a bone-shaped specimen and dimensional size. (**c**) universal tensile testing machine; (**d**) the geometry and size of tensile specimen.

The microstructure characteristics of TC4 and Al layers were investigated using EBSD (OXFORD, Nordlys Nano, London, UK). In detail, specimens were cut into 5 mm × 4 mm × 8 mm (length × width × thickness) from the TC4/Al_3_Ti MIL composites. Firstly, 2000- and 5000-grit silicon carbide sandpapers were used to grind the surfaces of the observed specimens, and 7000-grit sandpaper was used for final polishing and removing extremely fine imperfections to achieve a mirror-like surface. Then, 1.0 μm diamond paste was used for mechanical polishing. Afterwards, the Leica RES101 (Wetzlar, Germany) was employed to ion-etch the polished samples at 6.8 KV and 2.5 mA with a milling angle of 3. Additionally, the observed surface was examined on the YOZ plane (the coordinate system is shown in Figure 2a), with a step size of ~0.1 μm, and the data were post-processed using HKL Channel 5 software. Digital Image Correlation (DIC) was employed to investigate the distribution and evolution of local strain during the tensile test. The optimal speckle diameter was 0.1–0.3 mm, and a 30 × 30-pixel subset size was chosen. The ARAMIS^®^ post-processing program was used to calculate local strain.

### 2.3. In Situ and Quasi-Static Tensile Tests

Specimens for in situ SEM observation were sintered by stacking the thicker TC4 foils (~200 μm) and Al1060 foils (~150 μm), which were machined by wire-spark cutting to an observed length of 1.5 mm and a cross-section area of 2 × 5 (width × thickness, mm), as Figure 2b depicts. Then they were electropolished in a mixed solution containing 5 mL perchloric acid and 95 mL ethyl alcohol at 30 V for 80 s. The in situ tensile platform was installed in a Scanning Electron Microscope (SEM, Tescan Mira-3 LMF, Brno, The Czech Republic), which was used to conduct the in situ tensile test, as shown in Figure 2a. In situ tensile testing was performed at ambient temperature and a strain rate of 1 μm/s, and the tensile process was paused whenever SEM images were recorded at specific points.

The quasi-static tensile tests were conducted at a strain rate of 10^−3^/s on a GOTECH universal testing machine (Dongguan, China) (Figure 2c at ambient temperature, and the average tensile properties were determined from three independent tensile tests. As shown in Figure 2d, the bone-shaped specimens for uniaxial tensile tests were machined to an observed length of 18 mm and a cross-sectional area of 5 mm × 2 mm (width × thickness) using an electric spark wire-cutting machine. Additionally, the grip-section length was increased to ensure adequate friction because of the smooth surface of the outer TC4 layer, and aluminum foil was pasted on the specimen surface to prevent failure of the clamping section due to the brittleness of the Al_3_Ti phase.

## 3. Results and Discussion

### 3.1. Microstructure Characteristics

**Figure 3:**
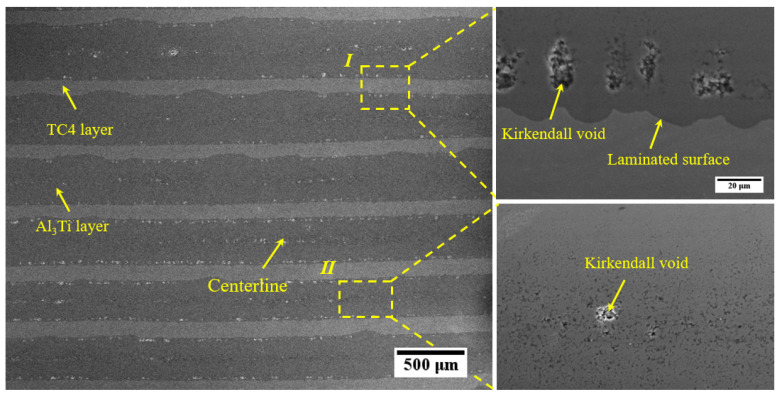
The typical SEM characterization of the TC4/Al_3_Ti MIL.

Figure 3 shows a typical SEM micrograph of the cross-section with multi-layer feature stacking by “TC4-Al_3_Ti-TC4” layers. It is clear that the thicker Al_3_Ti layers and the thinner TC4 layers are visible, while the Al layers have been completely reacted. As shown, the Kirkendall voids and centerlines are evident in the Al_3_Ti layers, attributed to oxide aggregations driven by the movement of the reaction front and the different diffusion rates of Ti and Al atoms [18]. Herein, the irregular shape of Kirkendall voids is distributed in the middle of Al_3_Ti layers and along the TC4/Al_3_Ti interfaces, which easily leads to stress concentration and induces microcrack nucleation when they gather together during the process of premature fracture failure. In addition, the increased dislocation density is beneficial for subsequent diffusion at the higher hot-pressing temperature, as reported in Ref. [16]. Thus, the distribution of Kirkendall voids and centerlines may enhance diffusion rates because their sizes are much larger than those of dislocations.

Moreover, the formation of the wavy TC4/Al_3_Ti interfaces is attributed to thermal expansion mismatch and shear deformation in the individual components. During the hot-pressing process, internal stress near the interfaces is generated to achieve synergistic deformation due to the varied Poisson ratios of the individual layers. Related research and theory can be referred to, such as the HR-DIC analysis as reported in [2,19,20]. Compared to the Al_3_Ti layer, the TC4 layer exhibits significant tensile ductility and undergoes more deformation during the hot-pressing process. Thus, in-plane tensile stress was induced in the Al_3_Ti layer, making it prone to tunnel crack formation. Therefore, it is also necessary to regulate the applied pressure at different stages to avoid microstructural defects, in addition to controlling the temperature and duration to ensure adequate reactive bonding between the TC4 and Al_3_Ti layers during the hot-pressing process.

### 3.2. Diffusion Kinetics and Reaction Process in the Ti-Al System

**Figure 4:**
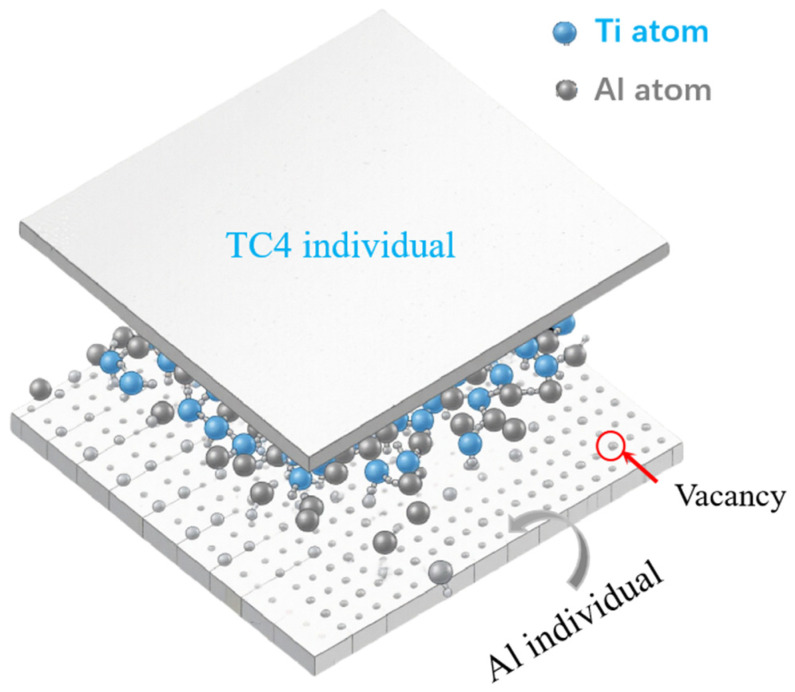
The schematic diagram of Ti and Al atomic diffusion kinetics and reaction.

During vacuum hot-pressing sintering, atomic interdiffusion and chemical reactions occur in the stacked TC4 and Al foils, and the Al_3_Ti phase forms at the reaction front at the TC4/Al interfaces [16]. As a result, the thickness of the TC4 and Al layers decreases, while that of the Al_3_Ti layer increases. Figure 4 shows a schematic diagram of Ti-Al atomic interdiffusion and reaction in the TC4/Al_3_Ti MIL. At the initial stage, Ti and Al atoms from individual layers diffuse into the intermixing zone, where they subsequently carry out a chemical reaction to form Al_3_Ti grains at the reaction front. As the interdiffusion process occurs by means of the path of grain boundaries and defects, Al atoms begin to react along Ti grain boundaries, leading to the nucleation of the Al_3_Ti phase [21]. Then the Ti and Al atoms must diffuse across the thicker Al_3_Ti layer, which makes the subsequent reaction kinetics dependent on the diffusion rates of Ti and Al atoms.

Furthermore, SEM observations reveal that Al_3_Ti particles exhibit a regular spherical morphology, suggesting that Al diffusion occurs preferentially along Ti grain boundaries in our previous papers [22]. Grain boundary diffusion is considered a faster pathway compared to bulk diffusion, enabling Ti and Al atoms from the constituent layers to diffuse and form solute segregation at Al_3_Ti grain boundaries. Such segregation predominantly occurs at triple junctions of larger Al_3_Ti grains, where smaller Al_3_Ti grains are observed surrounded by larger ones. As the heat treatment duration increases, the thickening of the Al_3_Ti layer impedes the diffusion of Ti and Al atoms, shifting the growth mechanism to diffusion-controlled kinetics. In this regime, the growth of the Al_3_Ti phase follows a parabolic characteristic [23]. Nucleation and growth of the Al_3_Ti phase then occur primarily at heterogeneous interfaces.

### 3.3. EBSD Characterization

**Figure 5:**
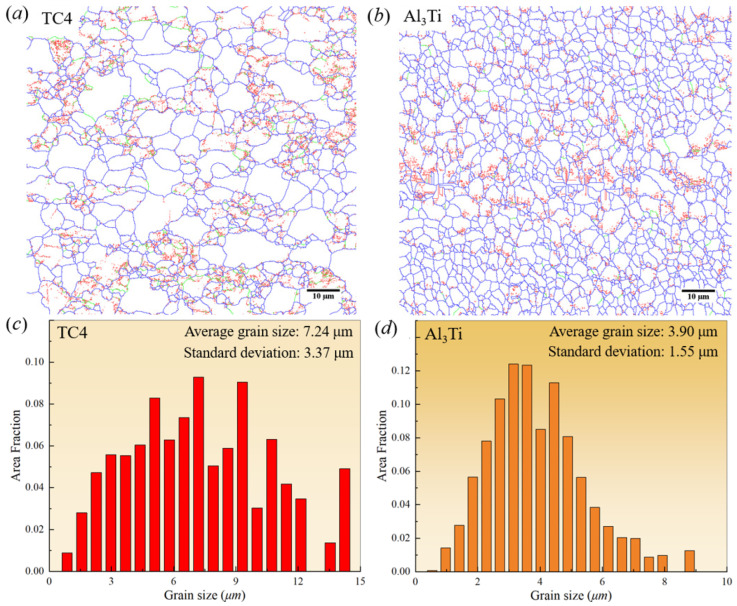
The distribution maps of grain boundary feature and grain size histograms for TC4/Al_3_Ti MIL. (**a**,**c**) TC4 individuals; (**b**,**d**) Al_3_Ti individuals.

To quantitatively evaluate the microstructure characteristics of TC4/Al_3_Ti MIL composites, Figure 5 shows the distribution maps of grain boundary features and grain size histograms for TC4 and Al_3_Ti layers, respectively. As shown in Figure 5a,b, the low-angle grain boundaries (LAGB, 2° < *θ* < 15°) are depicted by red and green lines, while blue lines represent high-angle grain boundaries (HAGB, *θ* > 15°). It is well established that the distribution of LAGB indicates the formation of sub-grains, which results from the rearrangement of dislocations into low-energy structures as dislocation density increases during hot-pressing deformation. According to the continuous dynamic recrystallization mechanism (CDRX) [24], if the rotational orientations of these sub-grains exhibit a large difference in value, these LAGBs would statistically transform into HAGBs without undergoing a nucleation process. In addition, these sub-grains may grow and/or merge through dislocation annihilation and rearrangement under prolonged exposure to the elevated hot-pressing temperature. As discussed in our previous work [22], grain refinement occurs in the TC4 layer during hot-pressing at 650 °C, driven by CDRX mechanisms, while the Al_3_Ti layer experiences grain growth at higher hot-pressing temperatures or longer hot-pressing times. Moreover, it can be seen that the equiaxed morphology of Al_3_Ti grains is presented, implying that Al atoms are mainly diffused along the grain boundaries, which is related to diffusion-controlled growth kinetics [25]. It is noted that the grain size of the Al_3_Ti layer increases as the distance from the interface increases [26], with a slight grain-size gradient. Therefore, both the grain size and boundary feature of TC4 and Al_3_Ti layers are significantly regulated by the hot-pressing temperature.

Based on the grain size statistical histograms for TC4/Al_3_Ti laminated materials shown in Figure 5c,d, the average grain sizes of the TC4 and Al_3_Ti layers are approximately 7.24 μm and 3.9 μm, respectively. Within the TC4 layer, the maximum and most frequently occurring grain sizes are 14.25 μm and 7.20 μm, accounting for approximately 4.91% and 9.29%, respectively. For the Al_3_Ti layer, these values are 8.80 μm and 3.15 μm, representing approximately 1.25% and 12.41% of the distribution, respectively. Additionally, the smaller average grain sizes in TC4 layers generally enhance tensile strength and uniform deformation, and the higher proportion of grain boundaries can significantly improve the work-hardening capacity of individual layers. Moreover, the constraint effects of grain rotation are imposed by neighboring grains; therefore, the statistical distribution of grains inevitably influences the mechanical properties of TC4/Al_3_Ti MIL composites. Consequently, stress concentration and delamination have readily occurred at TC4/Al_3_Ti interfaces, resulting from the varied grain sizes and deformation incompatibility between the TC4 and Al_3_Ti layers.

**Figure 6:**
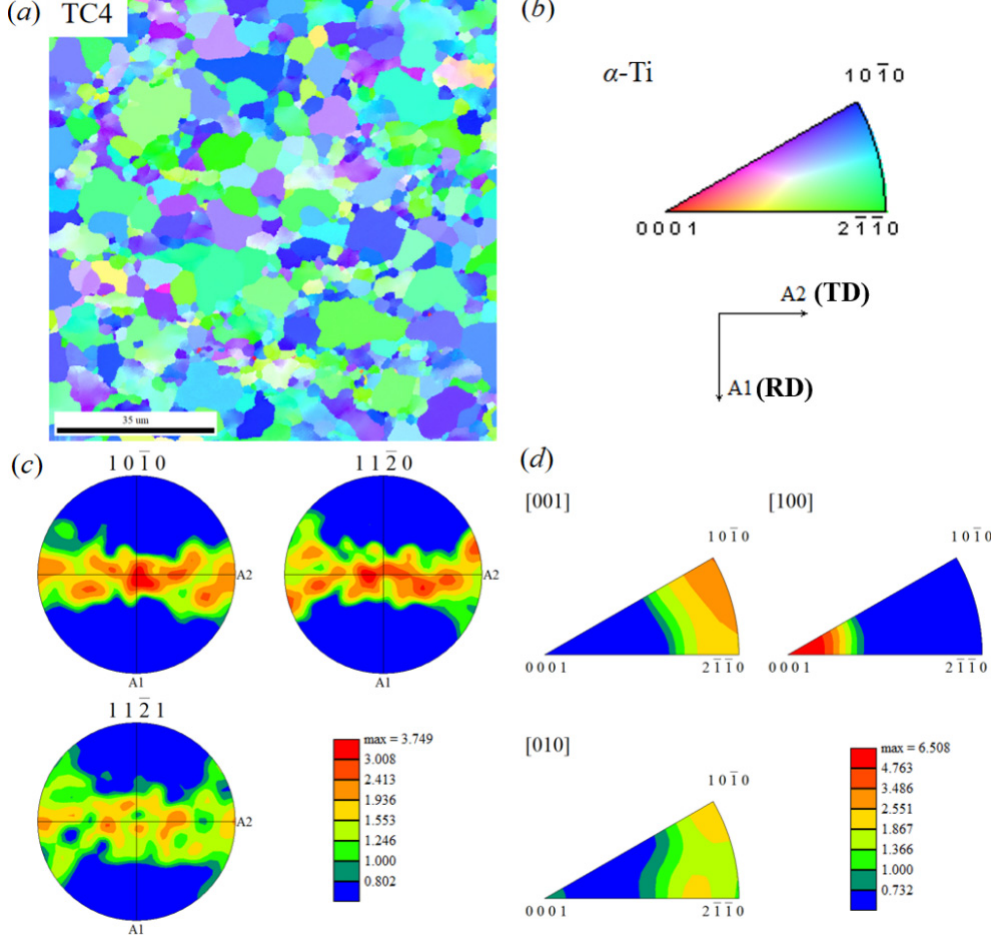
Texture characterization results of TC4 individual in TC4/Al_3_Ti MIL: (**a**) orientation image microscopy (OIM) map; (**b**) color code of TC4 phase; (**c**) pole figure (PF) of TC4 individual; (**d**) inverse pole figure (IPF) of TC4 individual.

**Figure 7:**
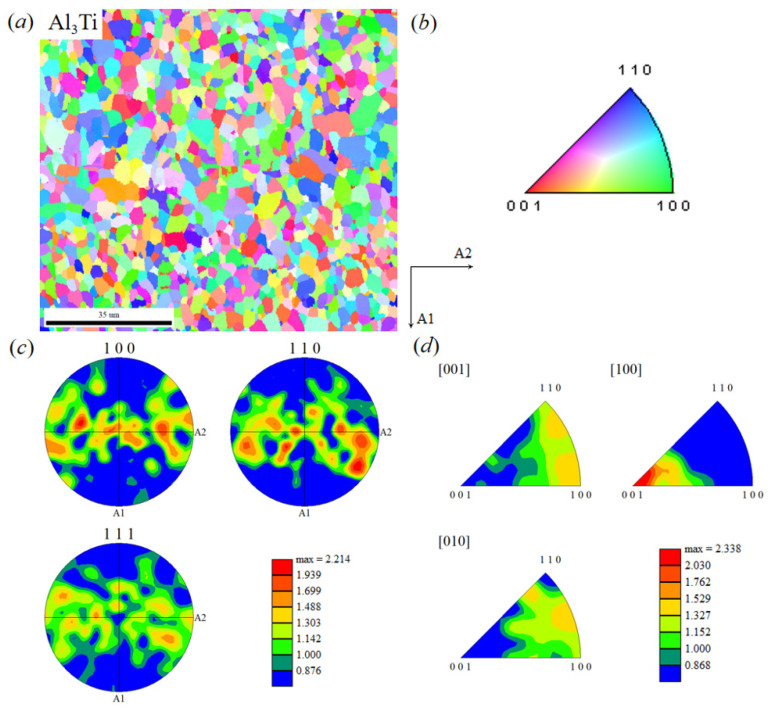
Texture characterization results of Al_3_Ti individual in TC4/Al_3_Ti MIL: (**a**) orientation image microscopy (OIM) map; (**b**) color code of Al_3_Ti phase; (**c**) pole figure (PF) of Al_3_Ti individual; (**d**) inverse pole figure (IPF) of Al_3_Ti individual.

As is well known, the mechanical properties of TC4/Al_3_Ti MIL composites are correlated with the crystallographic texture, which is respectively characterized for TC4 layers and Al_3_Ti layers in Figure 6 and Figure 7. Herein, Figure 6a shows the inverse pole figure (IPF) map of the *α*-Ti phase in the TC4 layer, where the colors representing grain orientations are defined by the orientation triangle shown in Figure 6b. The preferential distribution of specific colors provides an approximate indication of grain orientation. It can be observed that most grains appear predominantly in blue and green along the Normal Direction (ND) of the sample coordinate system, indicating that the grains within the selected region are mainly oriented along <10-10>//ND and <2-1-10>//ND. Combined analysis of pole figures and inverse pole figures in Figure 6c,d reveals high pole densities in the {10-10} and {11-20} crystallographic planes, with a maximum value of ~3.75. In the inverse pole figure, the <001> crystallographic direction exhibits a high pole density, with a maximum value of ~6.5. Further analysis based on standard pole-figure interpretation indicates that the texture in this region of the TC4 layer is a compound texture consisting of {10-10} <0010> and {11-20} <10-10>.

Similarly, as shown in Figure 7, the Al_3_Ti layer exhibits distinct fiber textures in the {100} and {110} crystallographic planes, with a maximum pole density of ~2.2. In contrast, the pole density distribution in the {111} plane is diffuse, indicating a very weak texture intensity. Within the {100} plane, regions of higher pole density are oriented at approximately ~10° to the transverse direction. Compared to the TC4 layer, the Al_3_Ti layer exhibits lower pole densities. This is primarily due to the original TC4 foil being in a rolled state and the TC4 layer’s texture in the TC4/Al_3_Ti laminate inheriting that of the original foil. The recrystallization, annealing, and dynamic recovery occurring during hot-pressing do not completely eliminate this initial texture. Additionally, the equiaxed Al_3_Ti grains likely possess a weaker texture. The results indicate that the Al_3_Ti layer exhibits {100} <001> and {110} <001> textures.

**Figure 8:**
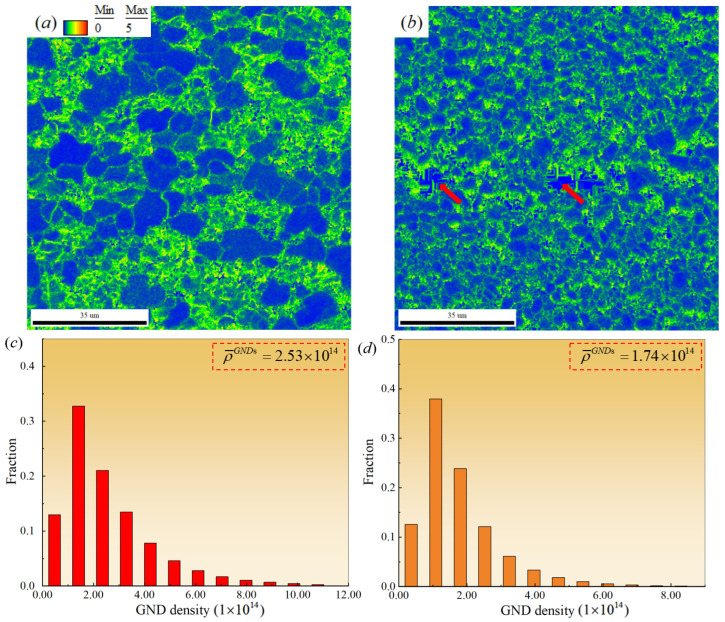
Kernel Average Misorientation (KAM) maps and statistical histograms for TC4/Al_3_Ti MIL. (**a**,**c**) TC4 individual; (**b**,**d**) Al_3_Ti individual.

To further elucidate the characteristics of grain misorientation and microstructural heterogeneity within the selected region of the TC4/Al_3_Ti MIL composites, the corresponding Kernel Average Misorientation (KAM) map and its statistical histogram were analyzed, as shown in Figure 8. The geometrically necessary dislocation (GND) density $\rho^{GNDs}$ and the average GND density ${\bar{\rho }}^{GNDs}$ can be calculated using the following formulas [22]:

(1) $$\rho^{GNDs}=\frac{2\theta }{\mu b}$$

(2) $${\bar{\rho }}^{GNDs}=\sum_{i=1}^{sum}\left(\rho_{i}^{GNDs}\times f_{i}\right)$$

where $\rho^{GNDs}$ is the density of GNDs, m^−2^; θ is the KAM value of the interest point (unit: rad); μ the unit length of the interest point; b is the size of the Burgers vector; $\rho_{i}^{GNDs}$ is the $\rho^{GNDs}$ at point ‘*i*’; and $f_{i}$ is the fraction of $\rho_{i}^{GNDs}$.

According to Figure 8a, high dislocation densities are primarily distributed along grain boundaries and within recrystallized grains in the TC4 layer. It is attributed that GND accumulation resulting from inhomogeneous deformation is prone to occur at interfaces and in Ti grains. When dislocation accumulation reaches a certain extent, tensile stresses develop near the piled-up regions, leading to microcracks or even cracking, which may cause premature failure of the material during service. Specifically, as the specimen deforms during hot-pressing, intragranular dislocations within the TC4 layer multiply, leading to a rapid increase in dislocation density and the formation of intertwined, entangled configurations. This impedes the movement of glide dislocations, thereby contributing to the work-hardening behavior of the heterogeneous structure. As dislocations continue to pile up within grains, the driving force for dynamic recrystallization increases gradually until the critical strain is reached [27]. At this stage, equiaxed recrystallized grains progressively replace the deformed grains, significantly reducing intragranular dislocation density and facilitating dynamic recovery. Thus, the evolution of dislocation configurations in the TC4 layer during hot-pressing is essentially governed by the competition between work hardening and dynamic recovery.

Figure 8b shows the KAM map of the Al_3_Ti layer. Compared to the TC4 layer, the uniformly fine grains in the Al_3_Ti layer are more prone to the wider GND distribution. These factors directly affect the deformation capability of the Al_3_Ti layer, making it highly susceptible to cracking at heterogeneous interfaces under significant external loads. Moreover, due to the presence of microstructural defects, such as pores and microcracks, in the Al_3_Ti layer, substantial stress concentration occurs, leading to continuous accumulation of residual strain. Consequently, dislocation density accumulation becomes more pronounced, and residual stress levels are higher. Based on the statistical results in Figure 8c,d, the average geometrically necessary dislocation density in the TC4 layer of the TC4/Al_3_Ti MIL composites is 2.53 × 10^14^ m^−2^, while that in the Al_3_Ti layer is 1.74 × 10^14^ m^−2^.

From a microscopic perspective, because the component layers of TC4/Al_3_Ti MIL composites (especially the TC4 layer) undergo grain rotation and changes in orientation under external forces, which significantly influence their plastic slip behavior, coordinated grain deformation occurs to maintain local deformation continuity. According to the Ashby model [28], additional local deformation gradients between grains geometrically necessitate dislocations to accommodate them. Under critical conditions, individual grains within the material locally initiate plastic deformation first. Therefore, the tensile behavior of TC4/Al_3_Ti MIL composites is related to the microscopic deformation behavior between grains and the macroscopic statistics of defect evolution. During their microscopic elastic–plastic deformation process, the dislocation migration within grains is significantly enhanced, and the density of mobile dislocations is markedly increased. It can be calculated using the Orowan Equations (3) and (4) as follows [29]:

(3) $${\dot{\gamma }}_{p}=\Phi (\rho_{m}+\Delta \rho_{m})b\upsilon$$

(4) $$\frac{\upsilon }{\upsilon_{0}}={\left(\frac{\tau }{\tau_{0}}\right)}^{m}$$

In Equation (3), ${\dot{\gamma }}_{p}$ is the shear strain rate, $\rho_{m}$ the dislocation density, $\Delta \rho_{m}$ the dislocation density variation, *b* the Burgers vector and *υ* the dislocation migration velocity. In Equation (4), *τ* is shear stress, *τ*_0_ the unit shear stress, *υ*_0_ the reference velocity at reference stress *τ*_0_, and *m* the stress sensitivity index.

Under constant-velocity tensile loading, the Burgers vector and shear strain rate of TC4/Al_3_Ti MIL composites can be regarded as approximately constant. According to Equation (3), the decreased dislocation migration velocity *υ* is the result of the increasing total dislocation density. Further, based on Equation (4), the dislocation migration velocity *υ* is proportional to the shear stress through a power-law relationship. Therefore, the plastic flow stress of the TC4/Al_3_Ti MIL composites ultimately decreases to the momentary tensile fracture, reflected in the lower tensile elongation in the following tensile test.

### 3.4. Tensile Properties and Strain Evolution

**Figure 9:**
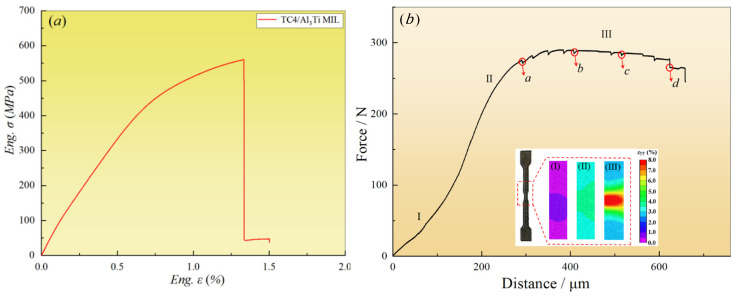
(**a**) The tensile engineering stress–strain curve. (**b**) The in situ load–displacement curve and the local strain evolution in the stage of plasticity. (Note: *a*~*d* attached to the position of the captured images are to stop the in situ tensile loading.)

The tensile engineering stress–strain curves for TC4/Al_3_Ti MIL composites are depicted by the red lines in Figure 9a. As can be seen, the tensile behavior is mainly regulated by the Al_3_Ti phase, showing no obvious tensile plastic stage since it contains a large volume fraction of Al_3_Ti layer and a thinner TC4 layer. In addition, the tensile test results show that the tensile strength of TC4/Al_3_Ti is 560.25 MPa, and the elongation is ~1.42%.

Figure 9b shows the in situ tensile load–displacement curve of the TC4/Al_3_Ti MIL composite, including elastic deformation (stage I), elastic–plastic deformation (stage II), and plastic deformation (stage III), following the isostrain condition. In stage I, the tensile deformation behavior presents the liner relation, wherein two straight lines with different slopes are related to the change in the cross-section of the sample. The subsequent stage II indicates the appearance of local damage, e.g., the initiation of microcracks and the growth of pores, which leads to a decrease in work-hardening ability. As tensile loading proceeds, stage III of plastic deformation is observed to form a stable plastic platform, and the jagged curve is attributed to stress relaxation when the load is held. It is noted that the plastic stage is not obvious (~350 μm) in the stress–strain curve of the TC4/Al_3_Ti MIL composite [30], and the load is mainly concentrated in the middle section of the specimens due to the smaller cross-sectional area. Therefore, the tensile properties of the in situ specimen are inferior to the stress–strain curve of a typical dog-bone specimen, and the final fracture location tends to occur at the observation cross-section of the specimen.

In addition, internal stress accumulates near heterogeneous interfaces under constraint effects due to mechanical incompatibility and synergistic deformation among individuals [5]. According to the Poisson effect [20], TC4 layers undertake compressive stress, and Al_3_Ti layers suffer tensile stress up to the occurrence of local failure. Meanwhile, microcracks initiate and propagate rapidly in Al_3_Ti layers, attributed to the tensile sensitivity of the Al_3_Ti phase. Consequently, delamination failure develops locally at TC4/Al_3_Ti interfaces, as verified by the following in situ tensile characterization. Consequently, the bearing capacity of the TC4/Al_3_Ti MIL composite is governed by crack propagation behavior, and the crack resistance is closely related to the toughening mechanism.

To study the local strain distribution during tensile deformation and the origin of failure at a strain rate of 10^−3^/s, the side surface of the specimen in the region marked by a red dotted line is characterized using Digital Image Correlation (DIC). As seen in stage I, the varied cross-section has little effect on the load distribution due to the lower macro strain, and the local strain distribution is uniform. As macro strain increases, the specimen’s strain intensity increases significantly in stage II. The strain path of MIL is found to be domain strain partitioning [31,32], and the elastic–plastic transition is induced by the mechanical incompatibilities of TC4 layers and Al_3_Ti layers. In stage III, the local strain is found to concentrate in the middle, and the deformation field is divided into several sub-regions, implying that a strain gradient is present. Moreover, it has been reported that the generation and accumulation of strain gradient contribute to hetero-deformation-induced (HDI) hardening near the interface-affected zone (IAZ), resulting in a superior combination of strength and ductility in MIL composites [33]. Moreover, the load-bearing capacity of TC4 layers mitigates premature brittle fracture of Al_3_Ti layers through stress-mediated strain transfer at the TC4/Al_3_Ti interface. Therefore, effective interface regulation and the generation of a strain gradient are of great significance for improving the mechanical properties of TC4/Al_3_Ti MIL composites.

### 3.5. In Situ Tensile Properties

**Figure 10:**
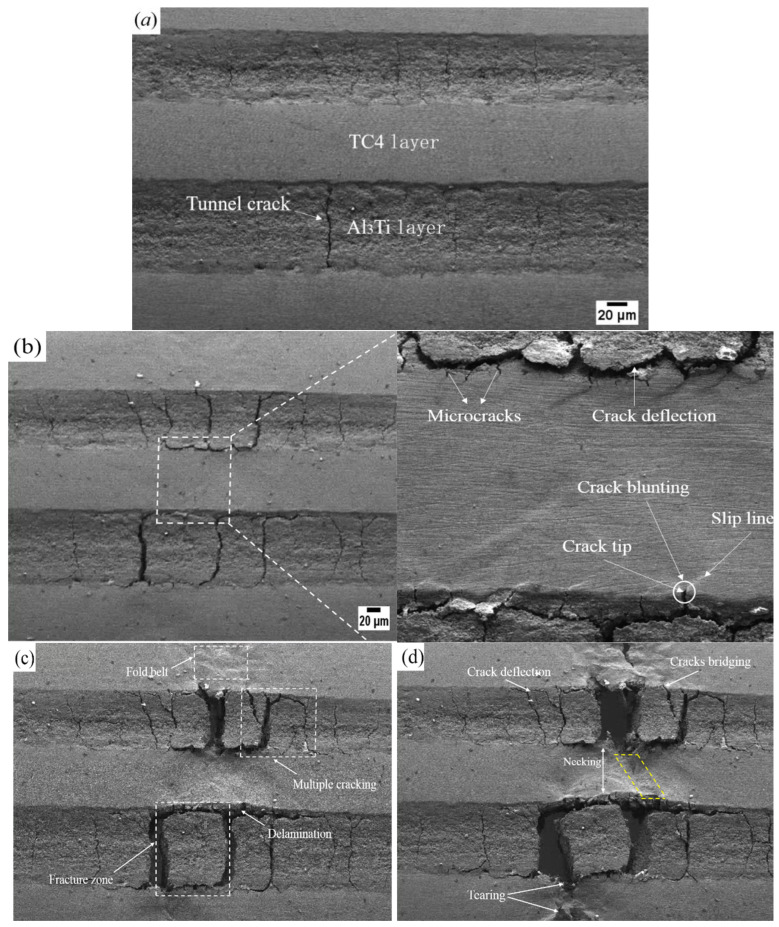
In situ SEM micrograph of crack propagation and fracture behavior. (**a**–**d**) The crack morphology corresponds to points (**a**–**d**) in the in situ tensile test.

As is well known, macroscopic deformation is always accompanied by microscopic crack growth, and in situ observation of crack propagation helps us better understand fracture behavior. The varied crack morphology observed in the in situ tensile test at plastic deformation (stage III) is shown in Figure 10. It can be seen that tunnel cracks were developed and penetrated through the overall Al_3_Ti layer in Figure 10a, which is perpendicular to the interface. And tunnel cracks, resulting from the coupling of thermal and tensile stresses [34], propagate faster than delamination. In addition, the geometric constraint imposed by adjacent ductile layers prevents further crack propagation, which is arrested at the TC4/Al_3_Ti interfaces due to a decrease in the crack-driving force. Thus, the TC4/Al_3_Ti MIL composite still possesses good bearing capacity when tunnel cracks form, as re-initiated crack propagation is driven by a larger driving force, consistent with Liu’s conclusion [35]. Therefore, the crack tolerance of TC4/Al_3_Ti MIL composites is enhanced by the laminated structure, compared to traditional metallic alloys.

Figure 10b shows that the perpendicular cracks become wider and more numerous, and extend throughout the Al_3_Ti layer as the tensile process proceeds. Meanwhile, the falling stage III in the load–displacement curve is caused by rapid crack propagation, which is attributed to cracks’ sensitivity to tensile stress. An enlarged view shows that microcracks have initiated and propagated in the TC4 layer, as evidenced by crack tips propagating towards the inside of the TC4 layer. As shown by the white circle, the slip line is nearly 45° to the tensile main stress trace in the TC4 layer, distributed along the direction of maximum shear stress. As reported, slip deformation permits stress concentrations to be redistributed, which avoids the occurrence of crack nucleation [36]. In addition, significant crack blunting occurs at the crack tip, which alleviates the sharp crack tip and reduces the stress concentration, thereby fully arresting the advancing cracks within the ductile layer. In this case, a larger crack propagation force related to external loading is required for a crack to re-nucleate, which equates to the increased toughness for TC4/Al_3_Ti MIL composites. In addition, there is another external toughening mechanism, crack deflection, as shown by the white arrow. It generally occurs when an advancing crack reaches the TC4/Al_3_Ti interface, which is found to reduce the local stress intensity by inducing cracks away from maximum stress [37]. Meanwhile, crack deflection implies the growth of the crack path, which significantly requires more surface energy. Therefore, the tendency toward crack instability is mainly reduced by extrinsic toughness mechanisms from the perspective of fracture behavior.

Figure 10c shows the fracture behavior at point c in the load–displacement curve. Based on analysis of the crack morphology, tunnel cracks in the Al_3_Ti layer are connected to the delamination propagation path, forming a block-like fracture zone, indicating a local failure mode of tensile fracture and interface delamination. According to the characterization results of the previous stage in Figure 10a, it can be seen that the tunnel cracks appear in the early tensile stage, and there is no obvious delamination at the TC4/Al_3_Ti interface. Therefore, the existing tunnel cracks facilitate the formation of a local fracture zone in the Al_3_Ti layer, and the crack propagating perpendicular to the layer thickness direction precedes and promotes the interface delamination. Meanwhile, the local deformation capacity of TC4/Al_3_Ti MIL is compensated by the formed cracks, and stress redistribution occurs to reduce the local stress. Moreover, a well-bonded interface remains in other regions. In other words, the closer to the fracture zone, the wider and longer the crack. Additionally, cracks nucleate and propagate continuously at stress concentrations. Thus, the crack density is increased, leading to multiple cracking, as depicted by the white dotted rectangles. It can be seen that the plastic deformation in the TC4 layer in the region of multiple cracking is more severe than that in the fracture zone, which is accompanied by the formation of a fold belt. Consequently, the occurrence of multiple cracking indicates that more plastic deformation and fracture energy are consumed, thereby enhancing the toughness of the TC4/Al_3_Ti MIL composite. It has been reported that local strain transfer behavior is enhanced by the TC4/Al_3_Ti heterogeneous interface [38], leading to more regions involved in plastic deformation and delocalizing microstrain, thereby alleviating stress concentration. Meanwhile, stress concentration at the interface leads to delamination and the formation of multiple cracks, thereby increasing the crack density. Clearly, the superior cracking tolerance and deformation stability against local failure are found in TC4/Al_3_Ti MIL composites with a well-bonded interface.

Figure 10d shows that the TC4/Al_3_Ti interface is nearly debonding in the fracture zone, which is examined by the rotation of the Al_3_Ti block. While the TC4/Al_3_Ti MIL composite is not entirely fractured, the increased load is found to be borne by the TC4 layers. As indicated by the white arrow, the obvious necking and tearing in the TC4 layer are developed. As shown, both necking and tearing are observed in the region depicted by the yellow dotted line. Meanwhile, significant tearing exists in the fold belt. It is indicated that the Al_3_Ti layers near the fracture surface have withdrawn from the synergistic deformation. Additionally, crack bridging occurs when unbroken Ti layers act as ligaments, stretching the Al_3_Ti cracks to reduce the crack-tip stress intensity. Therefore, TC4 layers are found to serve as efficient crack arresters, and crack propagation is retarded by the plastic tearing employed by ductile Ti layers. Therefore, the fracture resistance of the Al_3_Ti layer is significantly improved by the plastic deformation of the TC4 layers and the suppression effect on the crack tip.

## 4. Conclusions

TC4/Al_3_Ti metal–intermetallic laminated (MIL) composites were fabricated by the vacuum hot-pressing process, and the grain boundary distribution, crystallographic orientation and Kernel Average Misorientation (KAM) map were analyzed using EBSD. The distribution of local strain and crack-propagating behavior were characterized by DIC and in situ tensile experiments, respectively. The integration of EBSD, DIC, and in situ tensile techniques provides a robust framework for understanding the microstructure heterogeneity and fracture behavior in TC4/Al_3_Ti MIL composites, which are valuable for the design of lightweight, high-strength materials for aerospace and defense applications. The conclusions can be summarized as follows.

(1) The TC4/Al_3_Ti interfaces exhibit a well-bonded wavy morphology, with Kirkendall voids and centerlines predominantly located in the central region of the Al_3_Ti layer. These defects are attributed to the asymmetric diffusion rates between Ti and Al atoms during reactive sintering, which may serve as preferential sites for microcrack initiation under tensile loading.
(2) EBSD analysis reveals that the TC4 layer is characterized by a mixed texture of (10-10) ⟨0001⟩ and (11-20) ⟨10-10⟩, while the Al_3_Ti layer exhibits (100) ⟨001⟩ and (110) ⟨001⟩ fiber textures. The average geometrically necessary dislocation (GND) density in the TC4 layer (2.53 × 10^14^ m^−2^) is higher than that in the Al_3_Ti layer (1.74 × 10^14^ m^−2^), indicating a greater capacity for plastic deformation and work hardening in the ductile phase.
(3) Real-time observation of crack propagation reveals that tunnel cracks first initiate in the Al_3_Ti layer and propagate perpendicular to the interface, followed by interface delamination and plastic deformation in the TC4 layers. The primary extrinsic toughening mechanisms identified include crack deflection, crack blunting, crack bridging, multiple cracking, and plastic tearing of the ductile TC4 layers. These mechanisms collectively enhance the fracture resistance of the MIL composite by dissipating energy and reducing stress concentration at crack tips.
(4) DIC analysis shows that local strain progressively concentrates in the central region of the specimen during tensile loading, leading to the formation of a dominant fracture zone. The strain partitioning and gradient evolution near the TC4/Al_3_Ti interfaces contribute to hetero-deformation-induced (HDI) hardening, which plays a key role in delaying catastrophic failure.

## Figures and Tables

**Table 1:** Chemical compositions of raw TC4 foils and Al1060 foils.

Materials	Composites (wt %)
TC4	Ti: balance, Al: 5.5~6.8, Fe ≤ 0.30, V: 3.5~4.5, C ≤ 0.10, N ≤ 0.05, O ≤ 0.20
Al1060	Al ≥ 99.60, Si ≤ 0.25, Fe ≤ 0.35, Zn ≤ 0.05, Cu ≤ 0.05, V ≤ 0.05, Mn ≤ 0.03

## Data Availability

The original contributions presented in this study are included in the article. Further inquiries can be directed to the corresponding authors.
